# Case Report: Coil embolization of ascending aortic pseudoaneurysm in patient with Behcet's disease

**DOI:** 10.3389/fcvm.2024.1392236

**Published:** 2024-06-06

**Authors:** Mengyang Kang, Bo Zhang, Honggang Pang, Huishe Hu, Junbo Zhang

**Affiliations:** Department of Peripheral Vascular Diseases, The First Affiliated Hospital of Xi’an Jiaotong University, Xi’an, Shaanxi, China

**Keywords:** Behcet’s disease, ascending aortic pseudoaneurysm, coil embolization, thoracic endovascular repair, computed tomography angiography

## Abstract

**Background:**

Behcet's disease (BD) is a systematic vasculitis that affects vessels with various sizes, presenting as venous thrombosis and arterial pseudoaneurysms. The most severe manifestation in BD is ascending aortic pseudoaneurysm, which is associated with high risks of rupture and mortality.

**Case presentation:**

We present a case of ascending aortic pseudoaneurysm in a 50-year-old patient with BD. After preoperative evaluation, coil embolization was successfully performed to treat the pseudoaneurysm, resulting in a satisfactory outcome at the 1-year follow-up.

**Conclusion:**

Coil embolization serves as an effective treatment option for ascending aortic pseudoaneurysm in BD when open surgical repair and stent graft placement are unsuitable.

## 1 Introduction

Pseudoaneurysm of the ascending aorta represents a rare vascular complication in patients with Behcet's disease (BD) and carries a potentially fatal risk (1). Given the heightened threat of rupture and mortality, these individuals often necessitate either open surgical or endovascular repair. While traditional open surgical repair remains a viable option for select patients, the presence of anastomotic complications contributes to an unfavorable outcome, including issues such as leakage, occlusion, and pseudoaneurysm formation (2). Recently, mounting evidence has underscored the efficacy of endovascular repair (3, 4).

In this report, we present a case of ascending aortic pseudoaneurysm in a patient with BD, successfully managed through coil embolization, leading to a favorable outcome at the one-year follow-up. Consent was obtained from the patient for the publication of this data.

## 2 Case presentation

The patient, a 50-year-old man with a confirmed 16-year diagnosis of BD, was referred to our hospital because a recent computed tomography angiogram (CTA) conducted at another facility had shown a pseudoaneurysm of the ascending aorta. He had reported left-sided chest pain since 2 weeks prior. The patient was known with visual impairment, which was attributed to recurrent episodes of ocular inflammation, and over the last several years, he had experienced approximately 10–20 oral ulcers per year. Prior treatment with colchicine proved ineffective. Consequently, rheumatologists had adjusted the treatment regimen to incorporate glucocorticoids, cyclosporine, and adalimumab. However, during the tapering of adalimumab, the patient had experienced recurrent erythema nodosum in the extremities. The patient had discontinued adalimumab for 10 days upon admission to our center.

Additionally, the patient had comorbidities including hypertension and type II diabetes mellitus, for which long-term telmisartan 40 mg once daily and metformin 500 mg twice daily were prescribed. Upon admission, the patient reported mild left-sided chest pain, without accompanying compressive symptoms such as shortness of breath, dysphagia, stridor, or hoarseness. This subtle clinical presentation may be attributed to the pseudoaneurysm of the ascending aorta associated with BD, which can exhibit slow growth or even remain stable for a prolonged period, resulting in a lack of overt symptoms during chronic progression. The patient presented with a blood pressure of 117/53 mmHg and a heart rate of 65 beats/minute. A comprehensive physical examination revealed normal heart rhythm and apical position, absence of abnormal pulsations in the precordial region, and no pathological murmurs in the valve area. Electrocardiogram findings and biomarkers for cardiac injury were within normal ranges, excluding acute coronary syndrome.

**Figure 1:**
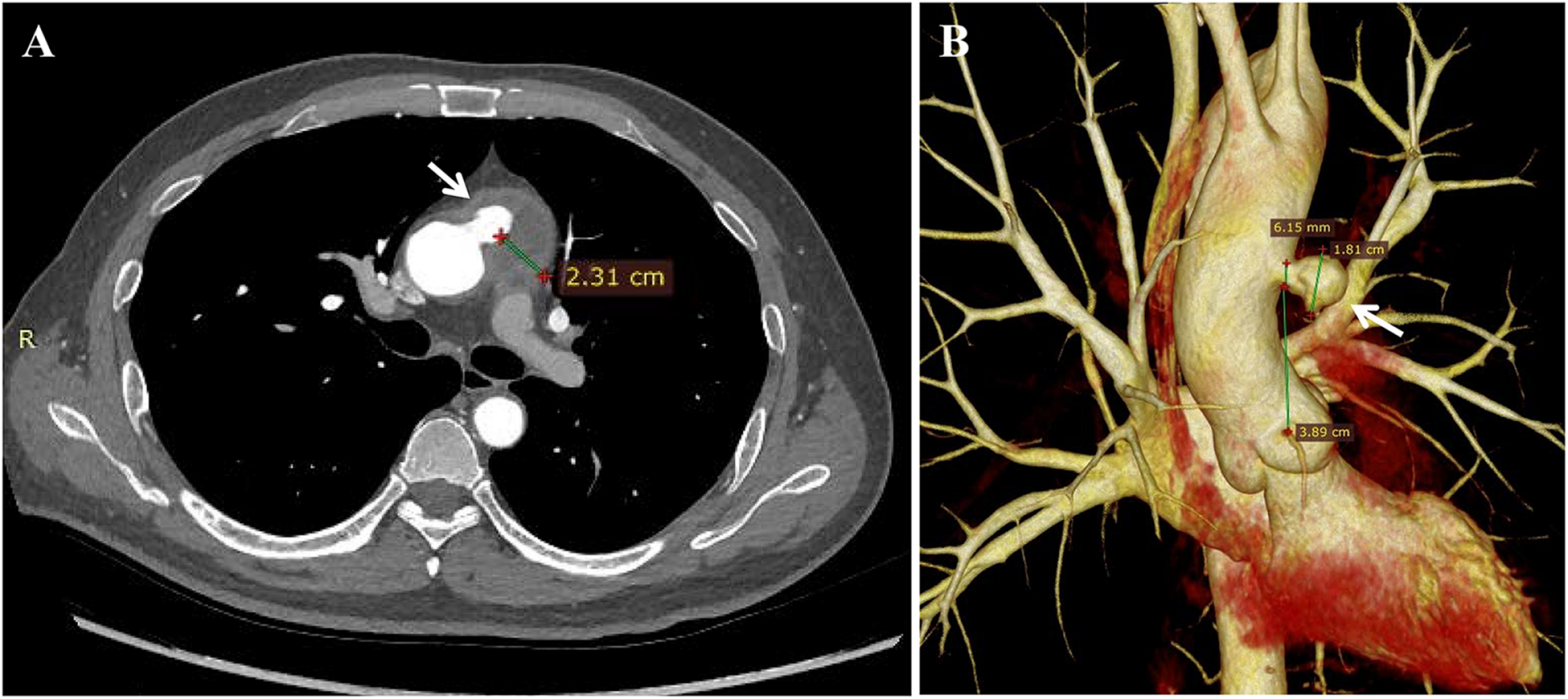
Preoperative CT angiography revealed (**A**) the pseudoaneurysm located at the anterior wall of the ascending aorta (indicated by the white arrow), with an adhered thrombus of maximum thickness 23 mm observed on its external surface. (**B**) Morphological measurements of the lesion were obtained from 3D reconstructed images, showing a maximum pseudoaneurysm neck diameter of 6 mm, maximum pseudoaneurysm sac diameter of 18 mm, and a distance of 39 mm from the aortic sinus to the pseudoaneurysm.

The patient had a hemoglobin level of 124 g/L (reference range 130–175 g/L), a high-sensitive C-reactive protein level of 8.08 mg/L (reference range 0–3 mg/L), and an erythrocyte sedimentation rate of 28 mm/h (reference range 0–15 mm/h). CTA revealed an ascending aortic pseudoaneurysm with a patchy hypodense opacity along the irregular wall of the aortic arch, indicative of an inflammatory condition. Preoperative CTA was used to evaluate morphology and size of the pseudoaneurysm. The aortic pseudoaneurysm was situated in the anterior wall of the ascending aorta, approximately 39 mm from the aortic sinus. The diameter of the neck of the pseudoaneurysm measured 6 mm, whereas its maximum sac diameter reached up to 18 mm, with an adhered thrombus with a maximum thickness of 23 mm observed on its external surface (Figure 1).

**Figure 2:**
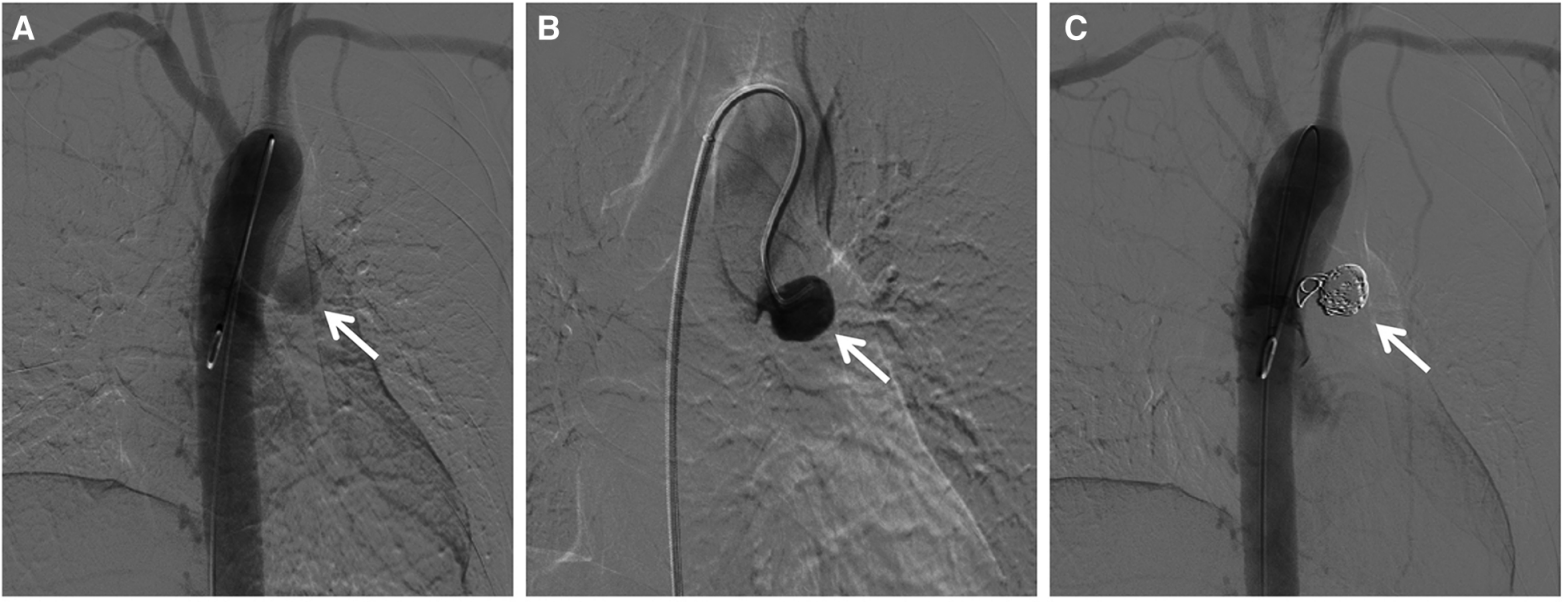
Intraoperative angiogram displayed (**A**) the anterior wall location of the aortic pseudoaneurysm and (**B**) clear visualization upon contrast administration. Following coil embolization, the angiogram demonstrated (**C**) complete occlusion of the pseudoaneurysm sac with coils, leading to exclusion from arterial blood flow (indicated by the white arrow).

**Figure 3:**
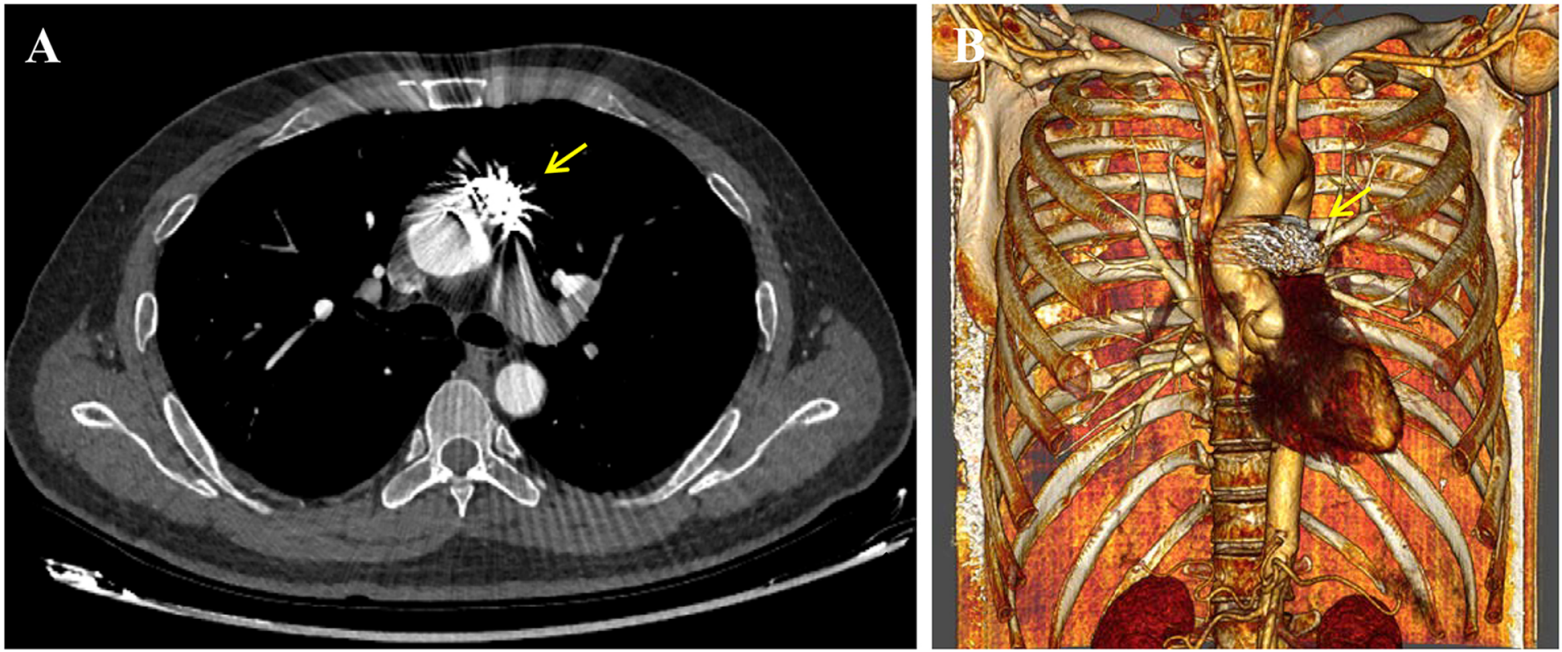
Postoperative CT angiography at the 1-year follow-up depicted (**A**) complete thrombosis and reduction in the size of the pseudoaneurysm sac. (**B**) 3D reconstructed image showed secure positioning of the coils without any evidence of migration (indicated by the yellow arrow).

**Figure 4:**
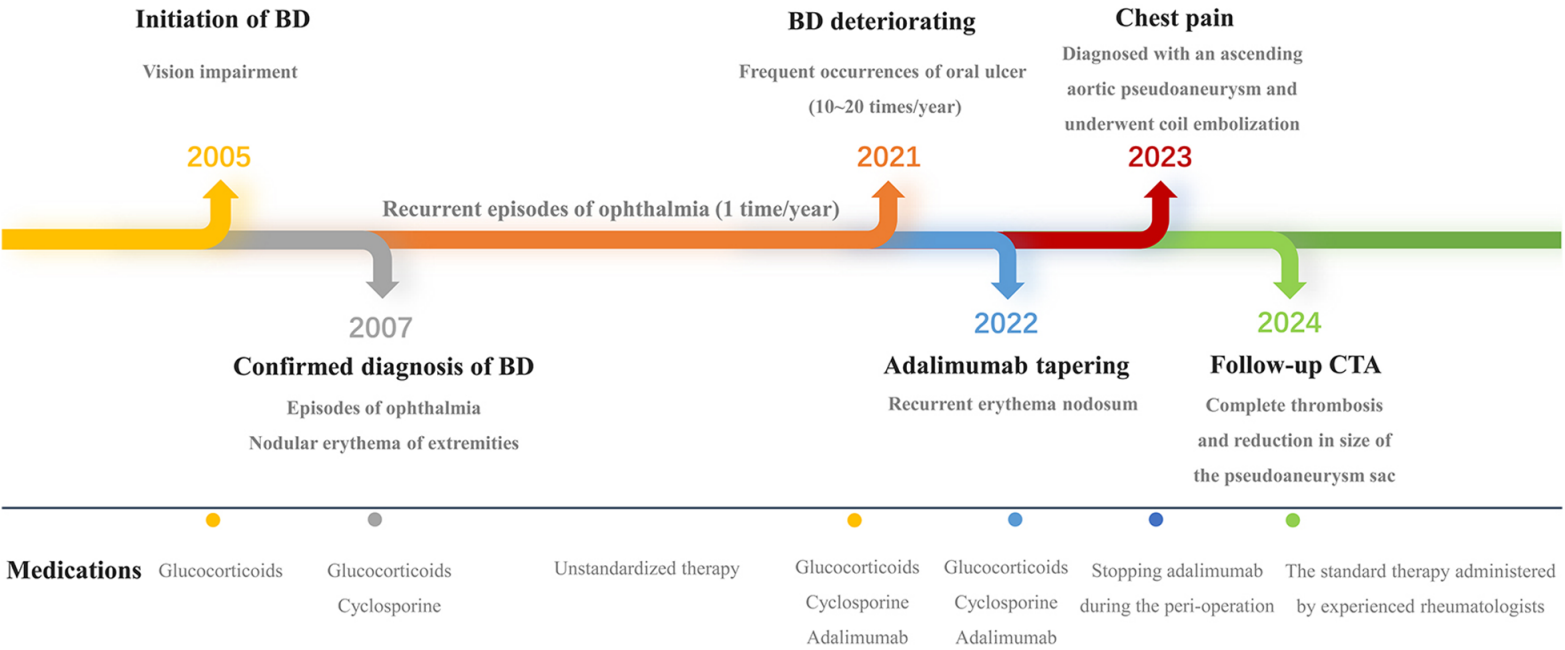
Timeline illustrating relevant data from the episode of care.

After multidisciplinary consultation, decision was made to perform coil embolization. Under local anesthesia, a pigtail catheter was introduced into the proximal end of the ascending aorta via the right femoral artery. Angiography revealed the presence of the pseudoaneurysm, located at the anterior wall of the aorta, with a maximum sac diameter of 20 mm, and a neck diameter of 6.7 mm. Subsequently, the pigtail catheter was replaced with a 5F multifunctional catheter (Terumo, Tokyo, Japan) enclosed in a 6F long sheath (COOK, Bloomington, IN, USA). Five Interlock coils of varying sizes were introduced into the pseudoaneurysm cavity, including one measuring 20–400 mm, two measuring 15–400 mm, and two measuring 10–250 mm. Intraoperative angiography confirmed complete occlusion of the pseudoaneurysm sac with coils, effectively excluding it from arterial blood flow (Figure 2). The procedure duration was 40 min. The patient remained admitted for 6 days and was subsequently discharged. Successful treatment was confirmed by a follow-up CTA scan 1-month postoperatively, which revealed no evidence of endoleak. Subsequent assessment by CTA 1-year postoperatively demonstrated complete thrombosis and reduction in the size of the pseudoaneurysm sac (Figure 3). Meanwhile, the erythrocyte sedimentation rate and high-sensitivity C-reactive protein levels remained within normal ranges with drug treatment. Currently, the patient is being followed up at regular intervals. Cardiac surgery will only be considered in case of occurrence serious, late postoperative complications (The timeline with relevant data from the episode of care is presented in Figure 4).

## 3 Discussion

In addition to oral and genital ulcerations, and relapsing ocular inflammation, BD also affects vessels, including venous thrombosis and arterial pseudoaneurysm (5). Ascending aortic involvement represents one of the most serious complications of BD, with aortic rupture emerging as the leading cause of mortality in patients with vascular involvement (1, 6). The pathogenesis of pseudoaneurysm formation in BD is associated with the occlusion of nutrient vessels within the arterial wall due to pan vasculitis. Chronic inflammation leads to proliferation of fibroblast and smooth muscle cells within the intimal layer, along with deposition in the arterial wall, resulting in destruction of the medial and outer layers. Pseudoaneurysms in patients with BD are highly prone to rupture and necessitate emergency open surgical or endovascular repair.

Open surgical repair has traditionally been considered the gold standard for treating ascending aortic pseudoaneurysms in patients with BD. However, this procedure is associated with high mortality rates and recurrent anastomotic pseudoaneurysms, leading to unfavorable outcomes. A meta-analysis demonstrated that the mortality rate following open surgical repair ranged from 10% to 30%, and 10% to 50% of patients with BD experienced recurrent pseudoaneurysms at the site of anastomosis (7). Moreover, because of the extensive trauma induced by sternotomy, and the deep hypothermic circulatory arrest, patients with major-comorbidities are often poor candidates for surgery.

Recently, endovascular repair has emerged as a novel option for treating pseudoaneurysms of the ascending aorta (3). The three most prevalent procedures include stent graft placement (8), vascular occlusion, and coil embolization (9), all of which have demonstrated reduced invasiveness and considerable efficiency. However, several unresolved questions hinder the widespread adoption of these interventions. Regarding stent graft placement, several challenges exist. Firstly, there is currently no stent graft specifically designed for ascending aortic lesions, limiting the options to off-label use of stent grafts designed for thoracic or abdominal aortic lesions. Secondly, the commercially available delivery systems used in our medical center are primarily designed for descending aortic lesions and may not provide sufficient length to effectively guide the stent graft from the femoral artery to the ascending aorta. Although using either the ventricular apex or axillary artery can reduce the delivery distance, it is unsuitable for patients with BD due to its high invasiveness (10). Lastly, mechanical stimulation and foreign body reactions caused by the stent graft may induce inflammation in the aortic wall (11), potentially leading to the recurrence of pseudoaneurysms at the edge of the stent graft. Several studies have also reported the successful use of endovascular occluders. Tarantini et al. (12) employed two Amplatzer septal occluder devices for percutaneous repair of ascending aortic pseudoaneurysm and aortopulmonary fistula. Komanapalli et al. (13) performed percutaneous repair of an ascending aortic pseudoaneurysm with a septal occluder device. When anatomically suitable, a less invasive approach using a vascular plug or septal occluder is an attractive option (14). However, the precise implantation of occluder devices at the neck of the pseudoaneurysm may pose technical challenges due to the variable location and size of these lesions. Currently, there is a lack of commercially available endovascular occluder devices in many medical centers. While coil embolization has become the preferred treatment for aneurysms in specific locations (15), its efficacy in the ascending aorta, particularly in patients with BD, remains uncertain. D'Ortenzio et al. (16) employed coil embolization to successfully address an aortic root pseudoaneurysm in a patient diagnosed with Loeys-Dietz syndrome. Dziekiewicz et al. (17) utilized coil embolization to treat a patient with post-traumatic pseudoaneurysm of the ascending aorta, demonstrating its efficacy as a safe and viable alternative for managing cases at high-risk for hemorrhage, where heparinization is unfeasible, both as a bridge procedure and as a long-term solution. Kim et al. (9) used coil embolization to address the pseudoaneurysm at the aortic sinotubular junction following multiple aortic operations and achieved a good outcome. Moreover, coil embolization can be used in combination with other endovascular techniques for the management of these patients. Wu et al. (3) combined bare stents with coil embolization to treat patients with vascular BD, resulting in an unsatisfactory outcome. Lyen et al. (18) utilized endovascular treatment to manage thoracic aortic pseudoaneurysms and proposed a novel treatment strategy that combined the use of occluder devices and coils as an effective treatment strategy for these patients. However, the efficacy of using coil embolization technology exclusively as an endovascular treatment option for ascending aortic pseudoaneurysm in patients with BD remains unclear.

To our knowledge, our procedure represents the pioneering use of coil embolization for treating ascending aortic pseudoaneurysms in patients with BD. The decision to employ coil embolization for managing this patient was based on several factors: firstly, the anatomical characteristics of an anterior wall location of the ascending aortic pseudoaneurysm with a narrow neck provided favorable endovascular conditions for coil embolization; secondly, considering the presence of BD in this patient, which renders the aortic wall more vulnerable to damage, we aimed to minimize iatrogenic injury caused by devices such as catheters and wires during the endovascular procedure. However, the utilization of coil embolization in ascending aortic pseudoaneurysms entails potential risks, including intraoperative aortic rupture and incomplete pseudoaneurysm filling, as well as postoperative endoleak and coil migration. To mitigate these adverse events, several key considerations were taken into account during the procedure: (i) gradual release of coils is essential to minimize the risk of pseudoaneurysm rupture; (ii) the combination of different sizes of coils can be employed to achieve complete filling and appropriate oversizing; (iii) precise positioning of coils is crucial to prevent distal migration, therefore we employed controlled-release coils that could be retracted multiple times, adjusted for correct orientation, and subsequently redeployed within the pseudoaneurysm cavity. Pseudoaneurysm at the puncture site is a commonly encountered complication following endovascular repair in patients with BD and vascular involvement. In this case, meticulous attention to the management of the puncture site led to the absence of post-operative pseudoaneurysm at the right femoral artery.

In strict accordance with the European League Against Rheumatism (EULAR) recommendations for the management of BD (19), the patient received regular immunosuppressant and glucocorticoid treatment pre-operatively, with coil embolization scheduled 2 weeks after adalimumab administration in this case, in order to mitigate the risk of postoperative complications such as enlargement or recurrence of pseudoaneurysm. Additionally, experienced rheumatologists adjusted perioperative and postoperative drug dosages to effectively control disease activity. The ESR and hsCRP levels remained within normal ranges during the 1-year postoperative period, indicating that this satisfactory outcome of coil embolization may also be attributed to the sustained administration of immunosuppressants. Therefore, we suggest that standardized continuation of immunosuppressive therapy is strongly recommended following endovascular treatment in patients with vascular BD. Many questions remain unaddressed regarding the long-term durability and safety of transcatheter coil embolization. Nevertheless, this technique seems to serve as a viable alternative for ascending aortic pseudoaneurysm in patients with BD, when open surgical repair or stent graft placement entails a significant risk of postoperative complications.

## 4 Conclusion

Ascending aortic pseudoaneurysm represents a rare and potentially fatal complication of BD. Coil embolization may present a viable alternative to sternotomy in patients with suitable anatomical characteristics.

## Data Availability

The original contributions presented in the study are included in the article/Supplementary Material, further inquiries can be directed to the corresponding author.
